# The Plague of 1348-50

**Published:** 1907-02-02

**Authors:** 


### THE PLAGUE OF 1348-50.

Recent accounts from India, Afghanistan, Mauritius, Hong-Kong, Japan, Russia, and Spain say that cases of plague have occurred. In India it is epidemic, but the outbreak at last appears to be on the wane. It is so long since the plague devastated England that the British public takes only a languid interest in the subject, and when the plague is spoken of reference is usually made to the outbreak which occurred in London in 1665. But even this visitation, great as it was, was dwarfed by the epidemic of 1603, and both sink into insignificance beside the awful years 1348-9, when the Black Death destroyed nearly a third of mankind and induced Brother John Clyn, of Kilkenny, to add a blank sheet of parchment to his chronicle "to continue it, if by chance anyone may be left in the future, and any child of Adam may escape this pestilence and continue the work thus commenced." The Black Death, as it came to be called after the Great Pestilences of 1603 and 1665, was pneumonic in character at the beginning, and was as fatal then as is this form at the present day. In the later stages, when the more resistant alone survived, it became chronic and of the ordinary bubonic type, with the usual lessened mortality. In London 200 bodies of the dead were buried daily in East Smithfield, Aldgate; and in the Charterhouse, where now is "High Level" in the playground of the Merchant Taylors' School, it is said that more than 50,000 persons were buried. This number may reasonably be looked upon as apocryphal, but the deaths were so numerous as to lead to the most serious economic crisis. The year 1348 found England mediæval; the year 1350 left her at the beginning of modern history. Religion no longer existed in its old magnificence of ritual, for the educated clergy had died and their place was taken perforce by those who could often do little more than read and write. The dreadful scenes of the previous years had made men deeply and personally religious; gilds became more numerous than ever, and out of the gilds soon came the great City companies. Whole tracts of country remained uncultivated, owing to the scarcity of labour, and then came the golden time of the poor. Statute after statute was passed to regulate the supply of labour and the rate of wages, but all without success. The labourer was triumphant, and labour began to understand its value and assert its power. The mediæval serfdom of England disappeared, the population was detached from the soil, and the old custom of holding land in small tenancies by a class of tenant proprietors gave place to a small body of men who held large tracts of land directly from the crown and farmed it on lease. The Black Death, too, is responsible to a large extent for the use of a common English language. French was the common talk of the upper classes throughout the country, but from the time of "the first Murrain this manner of spekyng was some dele y-changed," and the children of the nobility and gentry were taught their lessons in English, for the clergy who had hitherto acted as teachers and schoolmasters were dead, and there were but few learned persons to take their place. Even the very architecture changed, and long years often elapsed before the great church buildings in progress at the time of the plague were brought to a successful issue. It took England very many years to recover herself from the shock of the two-years' plague, and in the cases of some of the religious houses there is good reason to suppose that they never recovered themselves even to the time of the Reformation, when they were finally dissolved. Besides this Black Death all other plagues sink into insignificance. It seems to have been truly pandemic, whilst its predecessors and successors were fortunately epidemic. The reason for its wide distribution and great mortality appears to have been due to the pneumonic character of the outbreak, the pneumonia being, perhaps, associated with climatic conditions. The chronicle tells us that in 1348 it rained either by night or by day with hardly an exception from St. John the Baptist's Day (June 24) to Christmas; whilst in the Great Plague of 1665 it was a dry and warm year, with abundance of flies; and Samuel Pepys says that people were glad when the rains and frost came.

